# Folin-Ciocalteu Reaction Alternatives for Higher Polyphenol Quantitation in Colombian Passion Fruits

**DOI:** 10.1155/2021/8871301

**Published:** 2021-01-14

**Authors:** Juan C. Carmona-Hernandez, Gonzalo Taborda-Ocampo, Clara H. González-Correa

**Affiliations:** ^1^Grupo Investigación Médica, Universidad de Manizales, Manizales 170001, Colombia; ^2^Grupo Investigación Nutrición, Metabolismo y Seguridad Alimentaria (NUTRIMESA), Universidad de Caldas, Manizales 170004, Colombia; ^3^Grupo de Investigación en Cromatografía y Técnicas Afines (GICTA), Universidad de Caldas, Manizales 170004, Colombia

## Abstract

*Passiflora edulis* Flavicarpa, *Passiflora edulis* Sims, and *Passiflora ligularis* Juss are Colombian fruits (passion fruits) of important exportation value. They act efficiently as antioxidants, antifungal, and antimicrobial compounds due to their high polyphenol content. Polyphenols can be quantified by the Folin-Ciocalteu (F-C) reaction. Food matrices, solvent polarity, and several different reacting conditions are critical for the optimum extraction and quantification of polyphenols. Chromatographic identification and quantitation are satisfactory with access to a vast number of reference standards considering the availability of abundant phenolic compounds in crude extracts. The purpose of this study was to evaluate alternatives and specific F-C reacting conditions aiming at determining the highest total phenolic content (TPC) in three Colombian *Passifloras*. Among optimum reacting conditions, reduced reaction time and diluted alkali conditions yielded desirable positive results highlighting lower working time and minimum reagent waste production. For higher extraction yield, acetone 70% was the best solvent to capture more phenolics from the seedless pulp of these Colombian passion fruits.

## 1. Introduction

Fruits in the *Passifloraceae* family, mainly native to Central and South America, comprise over 450 different species [1, 2]. More than 80 edible *Passifloras* are cultivated throughout the world [3]. *Passiflora edulis* Flavicarpa, yellow passion fruit or maracuyá, is a popular and year-long harvested Colombian fruit. It is also produced, in a lower scale, in Australia, New Zealand, and Southeastern Asia [4]. This *Passiflora* offers several medical contributions, such as antioxidant, tranquilizer, and sedative, and it is normally consumed as fresh fruit, in juices, or as infusions [1]. Another less investigated fruit in this family is the *Passiflora edulis* Sims, known in Colombia as purple passion fruit or gulupa [5]. It has antioxidant, antifungal, and antimicrobial activities [6]. Colombia is one important gulupa exporter; in the year 2012, this Colombian *Passiflora* was the fruit with second best sales in international markets [7]. In the year 2017, the country exported more than 38,000 tons of gulupa [8].

In the same *Passifloraceae* genus, *Passiflora ligularis* Juss, commonly known as sweet granadilla (sweet passion fruit), is another nutritionally and commercially important Colombian fruit. With less annual harvesting production than gulupa and maracuyá, in 2017, granadilla production was more than 20,000 tons [8]. Different studies, in this *Passiflora*, are related to harvesting conditions, germination processes, and to its native and popular uses in local communities [9–11]. Still more studies in the seedless pulp of granadilla are needed in order to correlate total phenolic content (TPC) with its biological activities.

Phenolic compounds or polyphenols are plant secondary metabolites that offer medical and nutritional properties in regular diets [1, 7, 12]. Different extracting methods, such as solid-liquid, Soxhlet, ultrasound-assisted, microwave-assisted, and supercritical fluid extractions, are examples of common laboratory techniques aiming for the highest amount of polyphenols [12–15]. Some of these methodological approaches are time-consuming, expensive, and damaging to polyphenols due to hydrolytic or oxidative processes. Extraction alternatives, focusing on different food matrices with their special characteristics (seeds, flowers, leaves, fresh pulp), are recommended before attempting to measure optimum TPC values. For the last five decades, multiple studies report phenolic quantification with or without modifications in the Folin-Ciocalteu (F-C) method [16–19].

The Folin-Ciocalteu chemical process stresses in reduction-oxidation (redox) reactions, accomplished by electron transfer from phenolic groups to phosphomolybdic and phosphotungstic acid compounds, in an alkaline medium [16, 17]. Sodium carbonate (Na_2_CO_3_) is the alkali that extents an optimum pH value (of at least 10) for the proper reaction of phenolics with the F-C reagent [17]. This basic condition is essential for the ideal outcome in the F-C assay; meeting this criterion, phenolate ion groups are formed leading to the reduction of the acidic components in the F-C reagent [17, 19]. The reacting acids change from an initial light yellow to a blue color (reduced state) of different intensity based on the number of reacting phenolic groups [18, 19].

**Figure 1:**
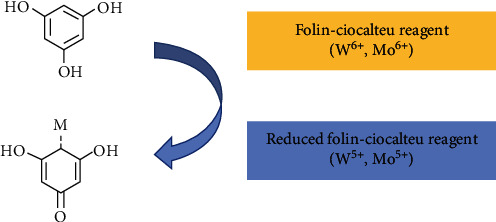
Chemical representation, color change, in the F-C reaction (W, tungsten; Mo, molybdenum reacting with polyphenol groups in an oxidation-reduction reaction) [16].

Figure 1 summarizes the F-C chemical process highlighting the redox reaction where the elements tungsten (W) and molybdenum (Mo) are reduced to an ionic form of +5, and the phenolic ring is oxidized. The presence of phenolic compounds is quantifiable, according to the degree of reduction of Mo^6+^ to Mo^5+^, yielding a blue color in the solution measured spectroscopically at 730 nm [19].

A high number of phytochemical compounds can undergo a redox process in the F-C assay. Reducing carbohydrates, ascorbic acid, aromatic amines, organic acids, and inorganic ions are examples of nonphenolic reacting compounds altering TPC based on the F-C method due to possible redox participation [17, 19]. The goal of this work was to evaluate lyophilization and extraction percentage yields in the seedless pulp of *P. edulis* Flavicarpa (Maracuyá, yellow passion fruit), *P. edulis* Sims (Gulupa, purple passion fruit), and *P. ligularis* Juss (Granadilla, sweet passion fruit) using three separately solvents, along with and without sonication aid. It is also aimed at exploring modifications in the F-C reaction looking for the highest total phenolic content in these three Colombian *Passifloras*.

## 2. Materials and Methods

### 2.1. Gathering (Geolocation) and Lyophilization of Fruit Pulp

All samples were collected from the Colombian departments of Valle del Cauca, Tolima, and Caldas. All fruit was picked at a ripeness stage of 5, optimum for human consumption [16]. Total fruit units were 50 to 70 expecting to gather at least 200 g of dehydrated pulp. Fruits were washed using water and 50 ppm sodium hypochlorite and dried with absorbent paper. The seedless pulp was collected and stored at -2°C [17].

Total seedless pulp content of each *Passiflora*, in portions of 40 g, was homogenized with 100 mL of distilled water in commercial blender. The homogenization product was centrifuged (Hermle Z 206 A, Wehingen, Germany) at 4000 rpm for 10 minutes at room temperature. The supernatants were placed in amber glass flasks and stored at 4°C for a later lyophilization process [18]. Lyophilization and freezing of the mature pulp were carried out to generate the main storage sample. The total pulp content, from each passion fruit, was lyophilized at 250 mTorr, (Virtis Genesis 25XL, Midland, ON-Canada), at a freezing rate of 0.5°C/min for 2000 minutes and 37°C as the final drying temperature. The lyophilized products were stored at -20°C in aluminum foil vacuum bags [17].

### 2.2. Extraction of Phenolic Compounds

The initial extraction process included a solid-liquid phase extraction with a total extraction time of 24 h. Lyophilized pulp samples, 0.5 g, were dissolved in 25 mL of ethanol (EtOH) 80%. The solution was stirred (Dragon Lab MS-H Pro, Beijing, China) for 30 min at 36°C and let stand for 24 h at room temperature. The alternative extraction processes, besides the solid-phase extraction, comprised other solvents and sonication. Samples of 0.5 g of each lyophilized *Passiflora* were placed in 25 mL of methanol (MeOH) 70% and acetone 70%, separately. The solutions were stirred for 30 min at 36°C and sonicated for 30 minutes (Branson series MH, mod. 3800, St. Louis, MO, USA). This extraction process lasted 24 hours. The extracts were centrifuged for 10 min at 3500 rpm. The supernatants were recovered for TPC quantification [19].

### 2.3. UHPLC-MS Analysis of Passion Fruit Polyphenols

Passion fruit extracts were dissolved in methanol : water (0.2% formic acid), vortexed, and sonicated for 5 minutes. All chromatographic analysis was accomplished in a UHPLC Dionex Ultimate 3000 (Thermo Scientific, Sunnyvale, CA - USA) equipped with a binary pump (HP G3400RS) and a Hypersil GOLD Aq (ThermoScientific, Sunnyvale, CA, USA) 100 x 2.1 mm, 1.9 *μ*m column at 30°C. Mobile phase A consisted of aqueous ammonium formate (0.2%) and B of acetonitrile with ammonium formate (0.2%). The initial gradient was set at 100% A switching linearly to 100% B over 8 min, then held at 100% B for 4 min before returning to 100% A in 1 min. Total run time was 13 min with 3 min postruns [20]. The identification was done with full-scan acquisition and ion extraction chromatogram (EIC) mode [M+H]^+^, and a precision of Δ_ppm_ < 0.001 using a mixed solution of external standards and comparable calibration curves (concentration range 0.05 to 5.00 *μ*g/mL).

### 2.4. UHPLC Reference Standard Compounds

Available comparable reference phenolics were caffeine, theobromine, theophylline, (±)-catechin, (-)-epigallocatechin gallate, (-)-epicatechin, (-)-epicatechin gallate, (-)-epigallocatechin, caffeic acid, *p*-coumaric acid, vanillic acid, rosmarinic acid, quercetin, naringenin, luteolin, kaempferol, ursolic acid, pinocembrin, carnosic acid, apigenin, ferulic acid, cyanidin-3-rutinosede, pelargonidin-3-glucoside, cyanidin, pelargonidin, quercetin 3-glucoside, and kaempferol 3-glucoside from Sigma Aldrich (St. Louis, MO, USA) [20].

### 2.5. Alternatives for Total Phenolic Content (TPC) Evaluation

Polyphenol content was determined colorimetrically based on the Folin-Ciocalteu (F-C) (Pan Reac Appli Chem, ITW Reagents, Darmstadt, Germany) reaction following procedures from Corrales-Bernal et al. (2015) with modifications. Alternatives for this reaction aimed for the highest detectable TPC in each extract. Adaptations in the F-C evaluation included assays with milli- and microvolumes of reactants, temperature changes, reaction time (30, 90, and 200 min), sodium carbonate concentration (20%, 10%, 7.0%, 3.5%, and 1.5%), and light exposure/protection. Samples of 1 mL of each extract were mixed with 1 mL of the F-C reagent (10%), shaken manually for 15 seconds, and mixed with 2 mL of 3.5% sodium carbonate (Na_2_CO_3_). The solutions reacted for 90 minutes [19]. All reactions were done in triplicates. A standard gallic acid curve was prepared as a comparative reference. Results are reported as mg of gallic acid equivalents per 100 g of fresh fruit (mg GAE/100 g FF). The evaluation of absorbance for TPC calculations was measured spectrophotometrically at 765 nm (UV/VIS Optizen POP®, Daejeon-South Korea).

### 2.6. Statistical Analysis

All results were evaluated considering the initial conditions of normality and homogeneity. Results were statistically tested for analysis of variance (ANOVA) followed by Tukey's test. Statistical significance for all data was set according to values for *p* < 0.05 [21]. The IBM SPSS Statistics software version v.20 (Armonk, NY, USA) was used for data analysis.

## 3. Results and Discussion

### 3.1. Geolocation and Lyophilization Percentage Gain

**Figure 2:**
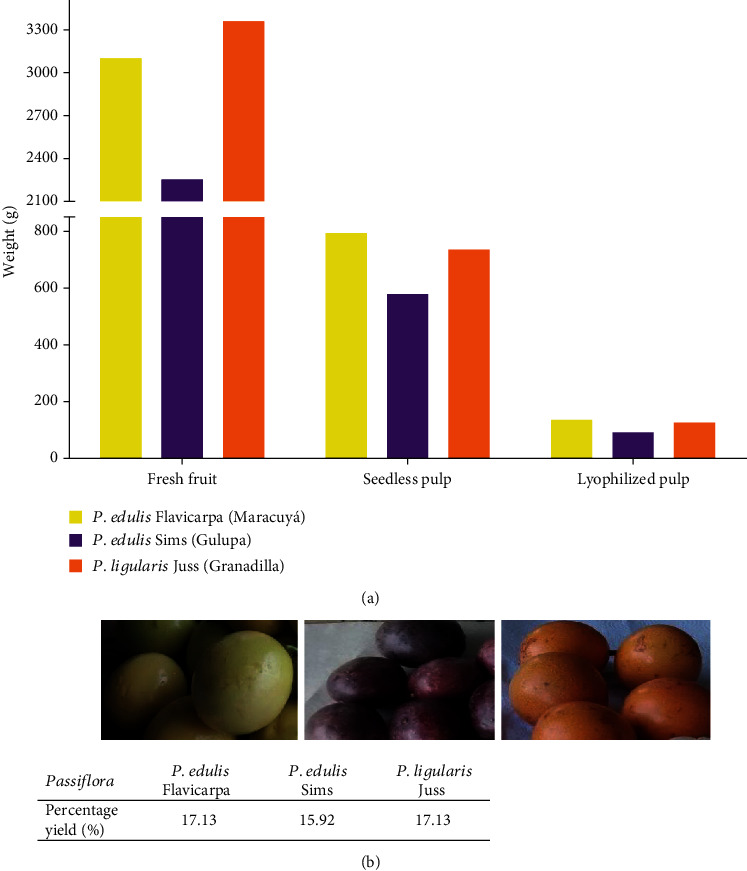
(a) Comparative weights of fruit treatment. (b) Pictures of fresh Colombian *Passifloras* and weight percentage yield after lyophilization.

The fruits of the genus *Passifloraceae* are harvested in different countries of the tropical and subtropical regions of the globe [22]. This study reports results for *Passifloras* cultivated in Colombia at altitudes between 1000 and 2000 meters above sea level (m.a.s.l.). *Passiflora edulis* Flavicarpa (maracuyá) was gathered in the municipality of Caicedonia-Valle del Cauca (1100 m.a.s.l, 4° 22′ 58.9^″^ N, 75° 48′ 42.1^″^ W, mean temperature 22°C), *P. edulis* Sims (Gulupa) in Cajamarca–Tolima (1814 m.a.s.l., 4° 26′ 30.5^″^ N, 75° 25′ 35.7^″^ W, mean temperature 19°C), and *P. ligularis* Juss (Granadilla) in Aranzazu–Caldas (1910 m.a.s.l., 5° 16′ 18.7^″^ N, 75° 29′ 25.0^″^ W, mean temperature 18°C).  Figure 2 shows initial, fresh weight, and final lyophilized amounts of the three *Passifloras*. The highest percent yield of extracts was registered for *P. edulis* Flavicarpa and *P. edulis* Juss. Mean humidity, in the fresh seedless pulp, of these Colombian *Passifloras* was about 84%.

Differences in percentage yield, for other studies done with fruits of the same family, coincide with results from the present study. An investigation using the fresh pulp of *P. edulis* Flavicarpa showed that its water content percentage was around 87% [23]. Other studies with comparable methodologies and fruits of the *Passiflora* species report similar differences with respect to percentage yield and total polyphenol content in *P. subpeltata* from India and *P. alata* Curtis from Brazil [2, 24].

### 3.2. UHPLC Peak Identification and Mass Spectra

Sixteen phenolic compounds were identified, via UHPLC/MS, in the *Passiflora* extracts including several other unidentified peaks.  Table 1 registers the presence/absence and mass spectrum response of identified phenolics based on the availability of 27 different reference standard compounds.

**Figure 3:**
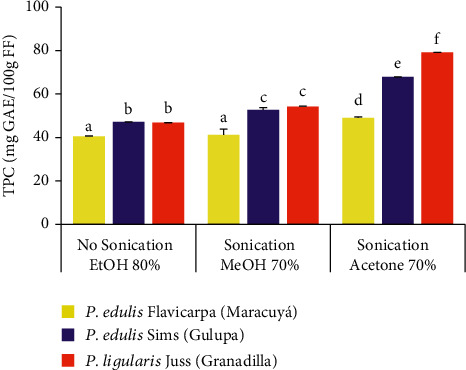
Comparative total phenolic content (TPC) in Colombian *Passifloras* extracted with different solvents and utilizing alternative extraction conditions. Different lower-case letters represent statistical differences, for means ± SD based (*n* = 3), according to ANOVA followed by Tukey's HSD test (*p* < 0.05).

UHPLC results in Table 1, according to the available reference standard compounds, yield the lowest concentration of phenolic compounds in *P. edulis* Sims (gulupa) 0.63 *μ*g/mL and the highest in *P. edulis* Flavicarpa (maracuyá) 1.70 *μ*g/mL. A very assertive chromatographic identification and quantitation relies on the disposition of a wide range of reference standards and on a recompilation of similar HPLC studies. This process is more time demanding and requires more investment. Alternative quantification processes for total phenolics can be evaluated based on spectrophotometric approaches, without the need for multiple standard reference compounds, and can be compared latter in this study as expressed in Figure 3 looking for a comparative quantification approach.

**Figure 4:**
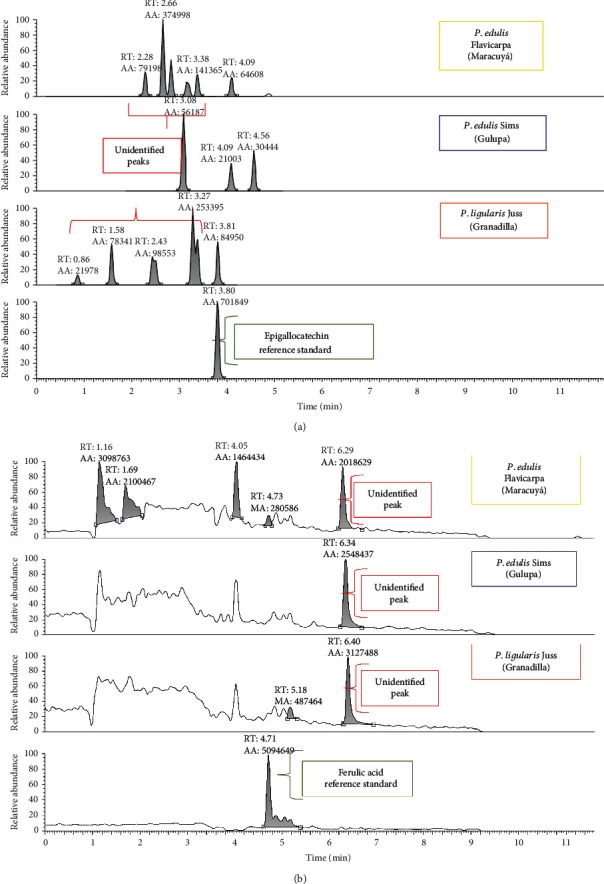
(a) Chromatograms for *Passiflora* acetone extracts with epigallocatechin reference standard. (b) Colombian *Passiflora* extracts with ferulic acid standard and several unidentified peaks in both graphs.

Other chromatographic studies coincide with several of the phenolic compounds found in this work; several researchers report the presence of other peaks representing phenolics found in passion fruits [25, 26]. These studies also show some unknown compounds and the presence of orientin, isoorientin, vitexin, and isovitexin as major polyphenols in several *Passifloras*; these four reference standards were not available for the present work. Figures 4(a) and 4(b) are graphical results highlighting the presence of unidentified comparable compounds detected in the *Passiflora* acetone extracts.

Results in this work show several unidentified compounds registering retention times between 1 and 3 minutes prior to the detection of the reference standard epigallocatechin found in the three Colombian *Passifloras*. A comparable study, using a more suitable number of reference standards, with similar chromatographic approaches reports the presence of the phenolics orientin, isoorientin, vitexin, and isovitexin [26]. These compounds registered later retention times as compared to the reference standard ferulic acid shown in Figure 4 coinciding with results in the present work where a major unidentified compound is registered at an approximate retention time of 6.5 minutes. These results suggest that some of the unidentified compounds might coincide with the ones already detected in other fruits of the same *Passiflora* family. The lack of a wider range of reference standards demands alternative and simpler quantitation processes such as the total polyphenol content (TPC) detected with the Folin-Ciocalteu method via spectrophotometric techniques. This colorimetric test tube assessment looks for the quantitation of a wider range of phenolics found in the passion fruit extracts. The Folin-Ciocalteu reaction detects all reacting phenolic groups undergoing oxidation-reduction processes leading to a more general quantification of phenolic and polyphenolic compounds.

### 3.3. Total Phenolic Content (TPC)

The evaluation of reagent volume, time of reaction, light exposure, alkali concentration, and temperature were considered for the modifications to the F-C method.  Table 2 shows reaction conditions aiming for the best phenolic quantitation in the *Passiflora* extracts in EtOH 80%. F-C reaction adjustments were focused on evaluating higher TPC values based on stoichiometry conditions, the avoidance of phenolate ion formation, and light interference that could affect the oxidation-reduction process or the interaction between reagents. The optimum reaction conditions were decided based on the statistical analysis (ANOVA followed by Tukey's HSD test) supporting the best treatment for each compared assessment.

The best TPC was decided after the evaluation and comparison of all alternative reacting assays according to several proposed reaction conditions. With respect to the amount of reactants, stoichiometry, or the relative quantities of F-C reagent and sodium carbonate, results were more considerable in the millivolume scale; a proportion of 1 : 2 yielded the best TPC outcome at a 90-minute reaction time. Doing the same reaction in the microvolume scale affected negatively the possibility of phenolic compounds to undergo a higher oxidation-reduction capability probably due to a more diluted medium taking into consideration the light interference for this reaction process.

A concentration of 3.5% sodium carbonate was the most appropriate proportion of alkali to react with the F-C reagent. Lower alkali concentration yielded lower TPC values, and higher concentrations of the alkali provided a more suitable medium for the formation of phenolate ions generating turbidity and affecting the spectrophotometric readings. Light and temperature for best TPC was considered due to the incidence of these variables in inducing higher oxidative stress in the reactions of phenolic compounds undergoing oxidation-reduction processes [27]. Keeping reactants protected for light exposure and working at a laboratory temperature of 17°C yielded optimum TPC values allowing reactants to interact more freely with the F-C components in an alkaline medium. The previous reacting conditions were necessary to establish the most appropriate assessment to extract the highest content of phenolic compounds from the pulp of the Colombian passion fruits.

Solid-liquid extractions of polyphenols from these *Passifloras* were achieved in two separate processes involving no sonication in the first set of assays and using EtOH 80% as the solvent. Sonication, along with solvents MeOH 70% and acetone 70%, was tested in the second assay. Initial TPC for *P. edulis* Flavicarpa (maracuyá), *P. edulis* Sims (gulupa), and *P. ligularis* Juss (granadilla) were, respectively, 40.50, 47.12, and 46.84 mg GAE/100 g FF.  Table 3 registers all TPC results considering different solvents used to capture the highest amount of available phenolic and polyphenolic compounds in the fruit extracts.

Due to the affinity of solvents for a wide range of phenolic groups based on their chemical composition (being both part of the polar and nonpolar compounds), acetone captured more phenolic compounds based on its amphiphilic condition [28]. Higher TPC was obtained, in the second process. The use of MeOH 70% and acetone 70% supported better solvent affinity for polyphenols in these extracts. Sonication aided in higher phenolic capture due to cell separation caused by ultrasound exposure and cell membrane rupture leading to the release of inner components [29]. Figure 3 displays comparative results for the extraction processes with applied modifications.

TPC improved using different solvents and a complementary sonication process. The solvent with the lowest effectiveness in the extraction of phenolic compounds was ethanol 80%. Methanol and acetone yielded better results; this outcome coincides with similar studies focusing on solvent-related phenolic extractions [30, 31]. Total phenolics in the fresh pulp of Colombian *Passifloras* maracuyá, gulupa, and granadilla, extracted with acetone 70%, were statistically different (yielding, respectively, *p* values of 0.017, 0.014, and 0.001) in comparison to phenolic compounds extracted with methanol 70%.

A study for the evaluation of TPC in *P. alata* Curtis showed that better results are solvent-related [24]. The classical Soxhlet extraction with ethanol and hexane yielded lower TPC, using ethanol [31]. Results in this previously reported investigation agree with the nonsonicated assays in the present work. Another research group, in a study with *P. subpeltata*, reported findings coinciding with our results. The use of methanol and acetone, for the extraction of polyphenols from the three *Passifloras*, yielded better results than ethanol [2].

Multiple studies (Table 4) report differences in the F-C method to measure polyphenols in plants. The volume of reactants and the concentration of Na_2_CO_3_ were significant factors for the optimum reaction outcome. The use of F-C : Na_2_CO_3_ (3.5%) in a proportion of 1 : 2 resulted in the best TPC values. This F-C approach also led to lower production of waste-reactants and pollution from the end-point of every reaction. Environmental aspects were considered since the F-C reagent produces hazardous decomposition compounds formed under fire conditions, along with sulphur, sodium, lithium, tungsten, molybdenum, and phosphorus oxides, hydrogen chloride gas, disodium wolframate dehydrate, phosphoric and hydrochloric acids, and lithium sulphate [32].

The extraction process modifications and the alternative F-C reaction conditions yield higher phenolic quantification in contrast with results in Table 1 for the calculated concentration of phenolics via UHPLC. Chromatographic results for the quantitation of phenolics in maracuyá were the highest. In this *Passiflora*, 6 major peaks were detected based on the comparison with the 27 available reference standards. For granadilla and gulupa, only 5 and 4, respectively, major peaks were found and quantified. These results suggest that for a general quantification of total polyphenol content in pulp of passion fruits, spectrophotometric approaches are a cheaper and a faster alternative.

## 4. Conclusions

In the case of these three similar Colombian fruits of the *Passiflora* species, *P. edulis* Flavicarpa (Passion fruit), *P. edulis* Sims (Gulupa), and *P. ligularis* Juss (Granadilla), the total quantification of polyphenols in lyophilized seedless pulp depends not only on the solvent type but also on the different reactive approaches regarding the optimum conditions in the F-C method. These considerations are relevant and applicable for cases where a throughout UHPLC analysis is not available for a lack of sufficient reference standards.

The best TPC resulted with acetone 70%, low proportions and volumes of F-C and Na_2_CO_3_ content (3.5%), no light exposure, room temperature, and a 90-minute reaction time. These results could be a model for future comparative studies, following the traditional F-C method with modifications, in which different biological actions of polyphenols are to be tested in fruits with similar characteristics.Additionally, alternate benefits were highlighted, such as the use of simpler and less expensive equipment (in comparison to microwave and supercritical fluid extraction instrumentation), low energy consumption, and minor production of reagent by-products and compounds with high polluting potential. Results in this work could contribute to environment-friendly chemical methods. It also supports less expensive and faster quantification reactions for polyphenols in high water content fruit pulp.

## Figures and Tables

**Table 1:** Standard reference compounds analyzed by UHPLC/MS and calculated polyphenol concentration in the three *Passiflora* acetone (70%) extracts. Compound found in extracts. LOQ, limit of quantitation 0.05 *μ*g/mL. LOD, limit of detection, concentrations below 0.02 *μ*g/mL.

Reference standards	Retention time (min)	[M+H]^+^ (m/z)	*P. edulis* Flavicarpa (Maracuyá)	*P. edulis* Sims (Gulupa)	*P. ligularis* Juss (Granadilla)
Theobromine	3.63	181.07109	0.09	<LOQ	<LOQ
Theophylline	3.78	181.07291	<LOD	<LOD	<LOD
Epigallocatechin	3.80	307.07966	<LOQ	<LOQ	0.09
Cyanidin 3-rutinoside	3.90	595.16072	0.60	<LOQ	<LOQ
(+)-catechin	3.97	291.08484	0.29	0.28	<LOQ
Pelargonidin 3-glucoside	4.06	433.11133	<LOD	<LOD	<LOD
Caffeine	4.09	195.08672	<LOQ	<LOQ	<LOQ
Caffeic acid	4.10	181.04859	<LOD	<LOD	<LOD
(-)-Epicatechin	4.13	291.08776	0.18	0.22	0.05
Epigallocatechin gallate	4.15	459.08990	<LOQ	<LOQ	0.07
Vanillic acid	4.16	169.04865	<LOD	<LOD	<LOD
Quercetin 3-glucoside	4.45	465.09987	0.09	<LOQ	<LOQ
Epicatechin gallate	4.49	443.09508	<LOD	<LOD	<LOD
Cyanidin	4.55	287.05416	<LOQ	<LOQ	<LOQ
Kaempferol 3-glucoside	4.59	449.10558	<LOD	<LOD	<LOD
p-Coumaric acid	4.63	165.05377	<LOQ	<LOQ	<LOQ
Quercetin	4.63	303.04838	<LOD	<LOD	<LOD
Ferulic acid	4.71	195.06422	0.45	<LOQ	0.54
Rosmarinic acid	4.78	361.08999	<LOQ	0.13	<LOQ
Pelargonidin	4.82	271.05928	<LOQ	<LOQ	<LOQ
Luteolin	5.24	287.05356	<LOQ	<LOQ	<LOQ
Naringenin	5.49	273.07433	<LOD	<LOD	<LOD
Apigenin	5.53	271.05874	<LOQ	<LOQ	<LOQ
Kaempferol	5.57	287.05644	<LOD	<LOD	<LOD
Pinocembrin	6.16	257.07951	<LOD	<LOD	<LOD
Carnosic acid	7.94	333.20433	<LOD	<LOD	<LOD
Ursolic acid	9.46	457.36631	<LOQ	<LOQ	<LOQ
Calculated concentration			1.70 *μ*g/mL	0.63 *μ*g/mL	0.75 *μ*g/mL

**Table 2:** Alternative F-C reacting conditions for TPC in *Passifloras* (∗ conditions representing significant differences and the best treatment based on ANOVA and post hoc Tukey's HSD test considering *p* < 0.05).

Reaction conditions	Compared variables	Observations
F-C (volume)	100 *μ*L, 1.0 mL∗	Using microvolumes (100 and 200 *μ*L led to higher variability in absorbance results and higher standard deviations).
Na_2_CO_3_ (volume)	200 *μ*L, 2.0 mL∗	
Na_2_CO_3_ (%)	1.5, 3.5∗, 7.0, 10, 20	Sodium carbonate concentration was determinant for the elimination of turbidity. The 3.5% assay avoided phenolate formation.
Time (min)	30, 90∗, 200	Reaction times at 30 and 200 minutes yielded no significant differences, and TPC values were higher at 90 minutes.
Light exposure	Yes/no∗	Exposure to light affected, at a higher level, reactions run in the micromilliliter level yielding higher variability.
Temperature (°C)	17∗, 36	Room temperature showed improved reaction results and higher TPC values.

**Table 3:** TPC in *Passifloras* extracted with three different solvents (∗best outcome and significant differences according to ANOVA and post hoc Tukey's HSD test, *p* < 0.05).

TPC in extracts (mg GAE/100 g FF)	*P. edulis* Flavicarpa (maracuyá)	*P. edulis* Sims (gulupa)	*P. ligularis* Juss (granadilla)
EtOH 80%	40.50	47.12	46.84
MeOH 70%	41.29	52.81	54.22
Acetone 70%	48.97^∗^	67.89^∗^	79.21^∗^

**Table 4:** F-C reaction parameters (reactants, concentrations, and reactant proportion) applied to different plant matrices (F-CR, Folin-Ciocalteu reagent).

Food matrix	F-CR (mL)	Na_2_CO_3_ (mL)	Reactant proportion	Reference
Fruit juice	2.5 (10%)	2.0 (7.5%)	2.5 : 2	[33]
Fresh fruit	6.0 (20%)	1.0 (7%)	6 : 1	[34]
Fresh fruit	2.5 (10%)	2.0 (7.5%)	2.5 : 2	[35]
Dehydrated fruit pulp	2.2 (10%)	1.0 (20%)	2.2 : 1	[36]
Lyophilized fruits and vegetables	10.0 (10%)	10.0 (7.0%)	1 : 1	[37]
Lyophilized leaves	2.0 (20%)	10.0 (10%)	2 : 1	[38]
Lyophilized whole plant	0.2 (20%)	0.2 (10%)	1 : 1	[39]

## Data Availability

The data used to support the findings of this study are available from the corresponding author upon request.

## References

[1] da Silva J. K., Cazarin C. B. B., Colomeu T. C. (2013). Antioxidant activity of aqueous extract of passion fruit (Passiflora edulis) leaves: in vitro and in vivo study. *Food Research International*.

[2] Saravanan S., Arunachalam K., Parimelazhagan T. (2014). Antioxidant, analgesic, anti-inflammatory and antipyretic effects of polyphenols from Passiflora subpeltata leaves – a promising species of Passiflora. *Industrial Crops and Products*.

[3] Arias-Suárez J. C., Ocampo-Pérez J. A., Urrea-Gómez R. (2014). La polinización natural en el maracuyá (Passiflora edulis f. flavicarpa Degener) como un servicio reproductivo y ecosistémico. *Agronomía Mesoamericana*.

[4] Ocampo J., Acosta-Barón N., Hernández-Fernández J. (2017). Variability and genetic structure of yellow passion fruit (Passiflora edulis f. flavicarpa Degener) in Colombia using microsatellite DNA markers. *Agronomía Colombiana*.

[5] Ángel-Coca C., Nates-Parra G., Ospina-Torres R., Melo Ortiz C. D., Amaya-Márquez M. (2011). Floral and reproductive biology of the ‘gulupa’ Passiflora edulis Sims f. edulis. *Caldasia*.

[6] Corrêa R. C. G., Peralta R. M., Haminiuk C. W. I., Maciel G. M., Bracht A. I., Ferreira C. F. R. (2016). The past decade findings related with nutritional composition, bioactive molecules and biotechnological applications of Passiflora spp. (passion fruit). *Trends in Food Science & Technology*.

[7] Bastidas D. A., Guerrero J. A., Wyckhuys K. (2013). Peticide residues in passifloras crops in regions of high production in Colombia. *Revista Colombiana de Química*.

[8] MinAgricultura (2018). "Agronet," Ministerios de Agricultura, Gobierno de Colombia. https://www.agronet.gov.co/Paginas/inicio.aspx.

[9] Amaya O. S., Devia E. H. V., Salamanca J. (2014). Prueba de extractos vegetales para el control de Dasiopsspp., en granadilla (Passiflora ligularis Juss.) en el Huila, Colombia. *Ciencia y Tecnología Agropecuaria*.

[10] Gutiérrez M. I., Miranda D., Cárdenas-Hernández J. F. (2011). Efecto de tratamientos pregerminativos sobre la germinación de semillas de gulupa (Passiflora edulis Sims.), granadilla (Passiflora ligularis Juss.) y cholupa (Passiflora maliformis L.). *Revista Colombiana de Ciencias Hortícolas*.

[11] Carvajal-de Pabón L. M., Turbay S., Álvarez L. M. (2014). Relationship between the folk uses of the granadilla plant (Passiflora ligularis Juss) and its phytochemical composition. *Biotecnología en el Sector Agropecuario y Agroindustrial*.

[12] Caldas T. W., Mazza K. E. L., Teles A. S. C. (2018). Phenolic compounds recovery from grape skin using conventional and non-conventional extraction methods. *Industrial Crops and Products*.

[13] Safdar M. N., Kausar T., Jabbar S., Mumtaz A., Ahad K., Saddozai A. A. (2017). Extraction and quantification of polyphenols from kinnow (Citrus reticulate L.) peel using ultrasound and maceration techniques. *Journal of Food and Drug Analysis*.

[14] Drosou C., Kyriakopoulou K., Bimpilas A., Tsimogiannis D., Krokida M. (2015). A comparative study on different extraction techniques to recover red grape pomace polyphenols from vinification byproducts. *Industrial Crops and Products*.

[15] Espada-Bellido E., Ferreiro-González M., Carrera C., Palma M., Barroso C. G., Barbero G. F. (2017). Optimization of the ultrasound-assisted extraction of anthocyanins and total phenolic compounds in mulberry (Morusnigra) pulp. *Food Chemistry*.

[16] Ford L., Theodoridou K., Sheldrake G. N., Walsh P. J. (2019). A critical review of analytical methods used for the chemical characterisation and quantification of phlorotannin compounds in brown seaweeds. *Phytochemical Analysis*.

[17] Sánchez-Rangel J. C., Benavides J., Heredia J. B., Cisneros-Zevallos L., Jacobo-Velázquez D. A. (2013). The Folin–Ciocalteu assay revisited: improvement of its specificity for total phenolic content determination. *Analytical Methods*.

[18] Rusak G., Komes D., Likić S., Horžić D., Kovač M. (2008). Phenolic content and antioxidative capacity of green and white tea extracts depending on extraction conditions and the solvent used. *Food Chemistry*.

[19] Chen L. Y., Cheng C. W., Liang J. Y. (2015). Effect of esterification condensation on the Folin–Ciocalteu method for the quantitative measurement of total phenols. *Food Chemistry*.

[20] Carmona-Hernandez J. C., Taborda-Ocampo G., Valdez J. C., Bolling B. W., González-Correa C. H. (2019). Polyphenol extracts from three Colombian Passifloras (passion fruits) prevent inflammation-induced barrier dysfunction of Caco-2 cells. *Molecules*.

[21] Moreno E., Ortiz B. L., Restrepo L. P. (2014). Total phenolic content and antioxidant activity of pulp extracts of six tropical fruits. *RevistaColombiana de Química*.

[22] Torres A. (2012). Caracterización física, química y compuestos bioactivos de pulpa madura de tomate de árbol (Cyphomandrabetacea) (Cav.) Sendtn. *Archivos Latinoamericanos de Nutrición*.

[23] Zapata K., Cortes F. B., Rojano B. A. (2013). Polifenoles y Actividad Antioxidante del Fruto de Guayaba Agria (Psidiumaraca). *Información Tecnológica*.

[24] Corrales-Bernal A., Vergara A. I., Rojano B., Yahia E., Maldonado M. E. (2015). Características nutricionales y antioxidantes de la uchuva colombiana (Physalys peruviana L.) en tres estadios de su maduración. *Archivos Latinoamericanos de Nutrición*.

[25] Shanmugam S., Gomes I. A., Denadai M. (2018). UHPLC-QqQ-MS/MS identification, quantification of polyphenols from Passiflora subpeltata fruit pulp and determination of nutritional, antioxidant, *α*-amylase and *α*-glucosidase key enzymes inhibition properties. *Food Research International*.

[26] Gomes S. V. F., Portugal L. A., dos Anjos J. P. (2017). Accelerated solvent extraction of phenolic compounds exploiting a Box-Behnken design and quantification of five flavonoids by HPLC-DAD in Passiflora species. *Microchemical Journal*.

[27] Pang H.-Y., Lee Y.-C., Wang G.-H., Liaw L.-L., Chen F. F., Chen Y.-P. (2020). Influence of light and temperature on secondary metabolites production by Monascus Ruber in rice solid cultures. *IOP Conference Series: Materials Science and Engineering*.

[28] Lakka A., Karageorgou I., Kaltsa O. (2019). Polyphenol extraction from Humulus lupulus (hop) using a neoteric glycerol/L-alanine deep eutectic solvent: optimisation, kinetics and the effect of ultrasound-assisted pretreatment. *AgriEngineering*.

[29] Annegowda H. V., Anwar L. N., Mordi M. N., Ramanathan S., Mansor S. M. (2010). Influence of sonication on the phenolic content and antioxidant activity of Terminalia catappa L. leaves. *Pharmacognosy Research*.

[30] Do Q. D., Angkawijaya A. E., Tran-Nguyen P. L. (2014). Effect of extraction solvent on total phenol content, total flavonoid content, and antioxidant activity of Limnophila aromatica. *Journal of Food and Drug Analysis*.

[31] Złotek U., Mikulska S., Nagajek M., Świeca M. (2016). The effect of different solvents and number of extraction steps on the polyphenol content and antioxidant capacity of basil leaves (Ocimumbasilicum L.) extracts. *Saudi Journal of Biological Sciences*.

[32] Everette J. D., Bryant Q. M., Green A. M., Abbey Y. A., Wangila G. W., Walker R. B. (2010). thorough study of reactivity of various compound classes towards the Folin-Ciocalteu reagent. *Journal of Agricultural and Food Chemistry*.

[33] Pereira M. G., Hamerski F., Andrade E. F., de Scheer A., Corazza M. L. (2017). Assessment of subcritical propane, ultrasound-assisted and Soxhlet extraction of oil from sweet passion fruit (*Passiflora alata* Curtis) seeds. *The Journal of Supercritical Fluids*.

[34] Ramaiya S. D., Bujang J. S., Zakaria M. H. (2014). Assessment of total phenolic, antioxidant, and antibacterial activities of *Passiflora* species. *Scientific World Journal*.

[35] Obregón García O. (2019). *Obtención de un alimento liofilizado a base de maracuyá (Passiflora edulis) y camucamu (Myrciariadubia)*.

[36] Chen G.-L., Chen S.-G., Zhao Y.-Y., Luo C.-X., Li J., Gao Y.-Q. (2014). Total phenolic contents of 33 fruits and their antioxidant capacities before and after in vitro digestion. *Industrial Crops & Products*.

[37] Sotelo D. I., Casas F. N., Camelo M. G. (2010). BOROJÓ (Borojoapatinoi): source of polyphenols with antimicrobial activity. *Vitae*.

[38] Díaz-García M. C., Obón J. M., Castellar M. R., Collado J., Alacid M. (2013). Quantification by UHPLC of total individual polyphenols in fruit juices. *Food Chemistry*.

[39] Chong C. H., Law C. L., Figiel A., Wojdyło A., Oziembłowski M. (2013). Colour, phenolic content and antioxidant capacity of some fruits dehydrated by a combination of different methods. *Food Chemistry*.

